# Unveiling the genomic landscape of possible metastatic malignant transformation of teratoma secondary to cisplatin-chemotherapy: a Tempus gene analysis-based case report literature review

**DOI:** 10.3389/fonc.2023.1192843

**Published:** 2023-06-22

**Authors:** Christian M. Farag, Elena K. Johnston, Ryan M. Antar, Shaher G. Issa, Qasim Gadiwalla, Zoon Tariq, Sun A. Kim, Michael J. Whalen

**Affiliations:** ^1^ Department of Medicine, George Washington University School of Medicine, Washington, DC, United States; ^2^ Department of Urology, George Washington University School of Medicine, Washington, DC, United States; ^3^ Department of Surgery, George Washington University School of Medicine, Washington, DC, United States; ^4^ Department of Pathology, George Washington University School of Medicine and Health Sciences, Washington, DC, United States

**Keywords:** cisplatin (CAS Number: 15663-27-1), malignant transformation of teratoma, chemotherapy resistance, TEMPUS, genetics, retroperitoneal, adenocarcinoma, liver metastasis

## Abstract

In this case report, we describe a patient who developed metastatic liver cancer of unknown primary origin one year following the surgical removal of a retroperitoneal adenocarcinoma. The retroperitoneal adenocarcinoma is considered a malignant transformation of teratoma (MTT), given the patient’s distant history of testicular tumor excised 25 years prior and treated with chemotherapy. Despite no primary tumor being identified, the leading primary hypothesis is that the liver metastasis stemmed from the resected retroperitoneal adenocarcinoma from one year prior. We theorize that the patient’s cisplatin-based chemotherapy 25 years ago may have triggered the MTT, as documented in the existing literature. Using TEMPUS gene testing on both the retroperitoneal adenocarcinoma and the recently discovered liver metastasis, we identified several genes with variants of unknown significance (VUS) that could potentially be linked to cisplatin chemotherapy resistance. While we cannot conclude that this patient definitively underwent MTT, it remains the most plausible explanation. Future research should investigate both the validity of the genes we have uncovered with respect to cisplatin resistance, as well as other genes associated with cisplatin resistance to further understand the pathogenesis of cisplatin resistance for better prediction of treatment response. As the world of medicine shifts towards individualized therapies and precision medicine, reporting and analyzing genetic mutations derived from tumors remains imperative. Our case report aims to contribute to the growing database of defined mutations and underscores the immense potential of genetic analysis in directing personalized treatment options.

## Introduction

Testicular carcinoma is the most common malignancy in young men, with 95% of cases comprising testicular germ cell tumors (TGCT) (1). The prognosis remains excellent among patients with localized and metastatic disease after appropriate multidisciplinary management.(2) Chemotherapy effectively treats metastatic testicular carcinoma, with a 5-year recurrence-free survival of approximately 75% (3). In rare cases, testicular cancer may reoccur after treatment, with most recurrences occurring within three years (4). Late recurrence beyond five - and even ten years - is rare and difficult to track, with the incidence reported to be about 1% of patients. Furthermore, recurrence multiple decades after primary resection is even more uncommon in the US, with few reports in the literature. Such delayed recurrence may implicate teratoma with malignant transformation instead of direct metastasis.

Although teratomas are generally benign, malignant teratoma (MTT) transformation can occur, likely due to somatic transformation into non-TGCT subtypes (e.g., rhabdomyosarcoma, adenocarcinoma) (5). The risk for malignant transformation increases with age, with squamous cell carcinoma being the most likely differentiation. Additionally, some studies propose that the incidence of MTT increases with exposure to chemotherapy - which eradicates chemo-sensitive cells - leaving chemo-resistant cells to continue growing (6, 7).

Currently, there is a paucity of literature describing clinicopathologic or genetic predictors of MTT. In this case, we present a 43-year-old man who presents with retroperitoneal adenocarcinoma two decades after radical orchiectomy and systemic chemotherapy for testicular cancer and develops metastatic cancer to the liver of unknown primary. Through this case’s tumor pathology, molecular profiling, and literature review, we postulate a genetic diathesis for cisplatin-resistance, prompting metachronous malignant transformation and subsequent metastasis.

## Case

The patient is a 43-year-old male who presented with a two-week history of progressive left-lower-quadrant and testicular pain. Medical history included hypertension and testicular cancer, status-post right orchiectomy, and chemotherapy ~25-30 years ago. Treatment for his testicular cancer took place in Central America, with details such as exact TNM-Stage unclear, but the patient endorses a history consistent with cN+ and an unknown chemotherapy regimen.

Before the patient’s first presentation to our institution in November 2021, he had been hospitalized three months prior for similar symptoms, and demonstrated a 5-cm periaortic cystic mass. Fine-needle aspiration was performed then, cytology demonstrated atypical cells only, and CT-Chest was negative for any nodules. However, the patient was unfortunately lost to follow-up.

Upon presenting in November 2021, the patient underwent scrotal ultrasound without evidence of a left testicular mass. The patient then underwent FDG-PET scan, which demonstrated a 5-cm hypermetabolic retroperitoneal complex cystic mass suspicious for malignancy or disease recurrence given the patient’s known history of testicular cancer. After thorough counseling and informed consent, the patient was taken to the operating room for exploratory laparotomy, excision of the left retroperitoneal mass, and left retroperitoneal lymph node dissection. There was a severe desmoplastic/fibrotic reaction around the tumor, cementing it to the underlying vena cava and aorta, necessitating intraoperative vascular surgery consultation for complete resection. The inferior mesenteric artery was also encased by the tumor and was resected en-bloc with the mass. Para-aortic and preaortic lymph nodes were removed as part of the mass. Manual and visual inspection of the peritoneal cavity revealed no evidence of metastatic disease to the liver or small bowel. The patient was then scheduled to complete chemotherapy but was lost to follow up again.

**Figure 1:**
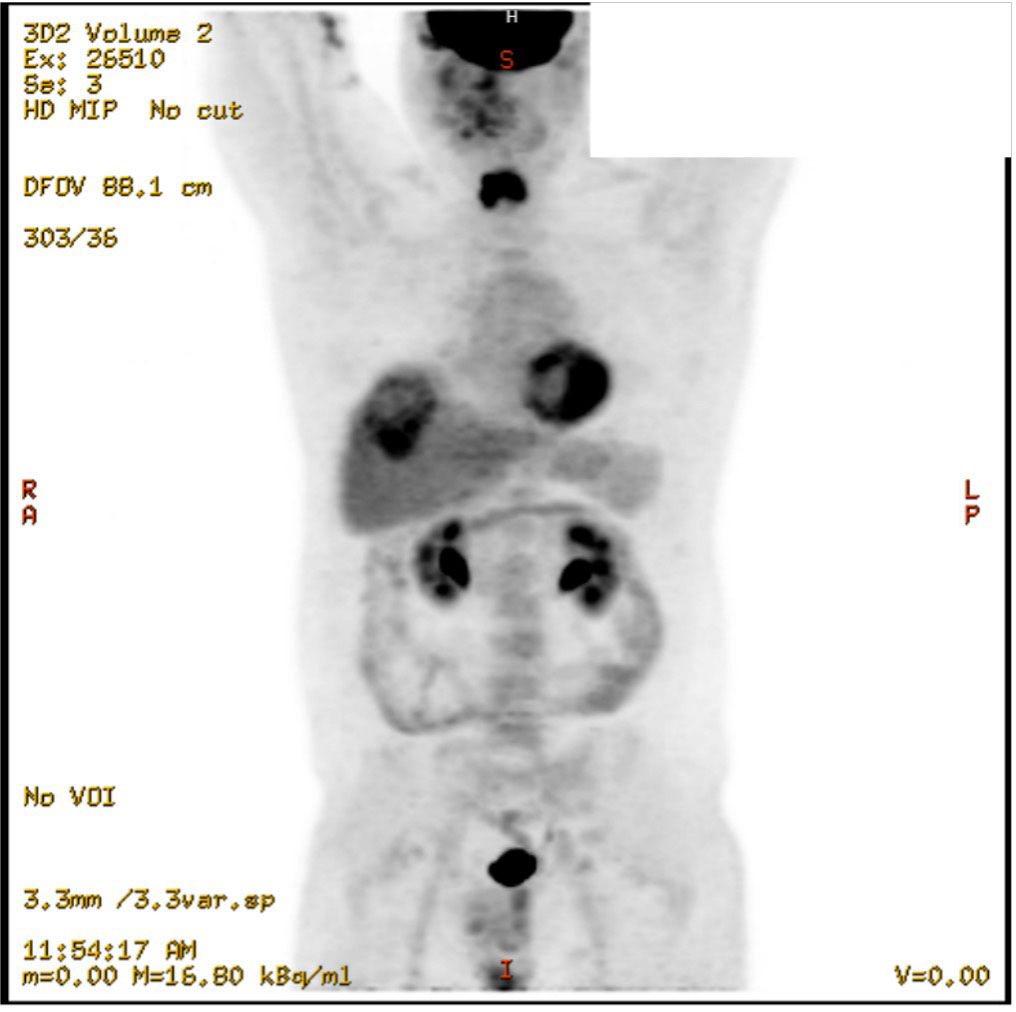
Coronal view of PET scan, showing the 6cm hepatic mass and no clear primary tumor to explain metastasis, December 2022.

One year later in December 2022, the patient presented to the emergency department at our institution with midline lower back pain radiating to the left groin. The pain was attributed to a surgical clip. However, imaging revealed an incidental indeterminate 6-cm hypodense hepatic lesion in segment 8 with an adjacent and inferior 1.9 cm indeterminate lesion, representing a possible small satellite lesion. Tumor markers (AFP, LDH, b-HCG, CEA, CA 19-9) were all negative. An ultrasound-guided liver biopsy was performed (see pathology results below), and the patient was discharged with outpatient follow-up instructions. In outpatient, the patient underwent PET scan, which did not identify a primary site of malignancy (**Figure 1**). The patient was ultimately diagnosed with Stage IV cancer of unknown primary with metastatic cancer to the liver and started on six cycles of paclitaxel for palliative management.

**Figure 2:**
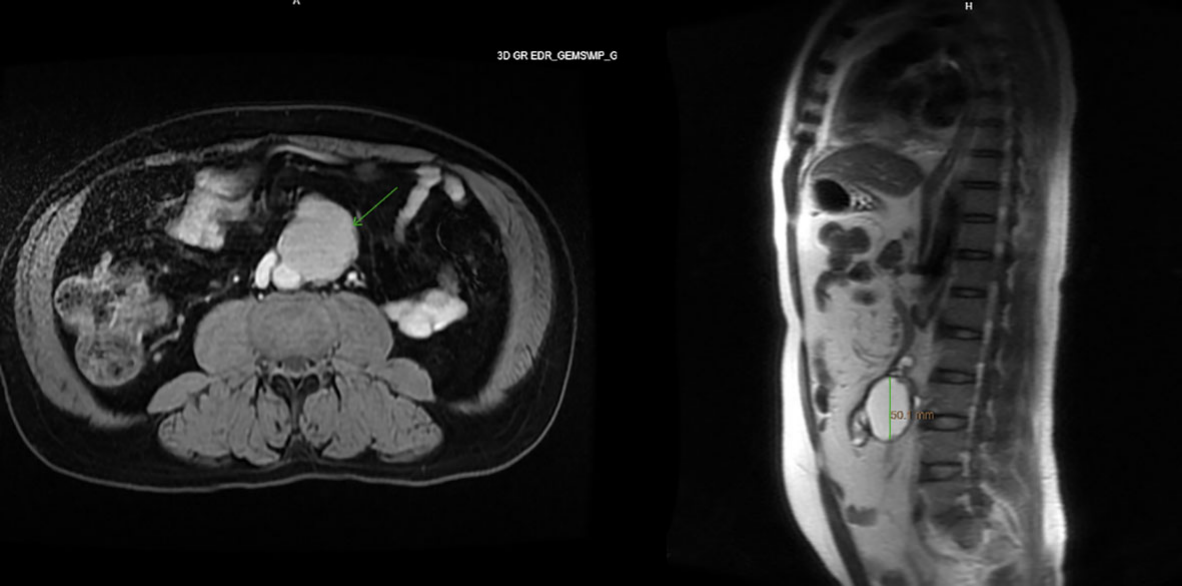
Axial and sagittal view of MRI abdomen, showing a 5.4 x 5.1 x 4.1cm T1 and T2 hyperintense lesion with areas of nodularity and thick septation along the anterior edge, November 2021.

**Figure 3:**
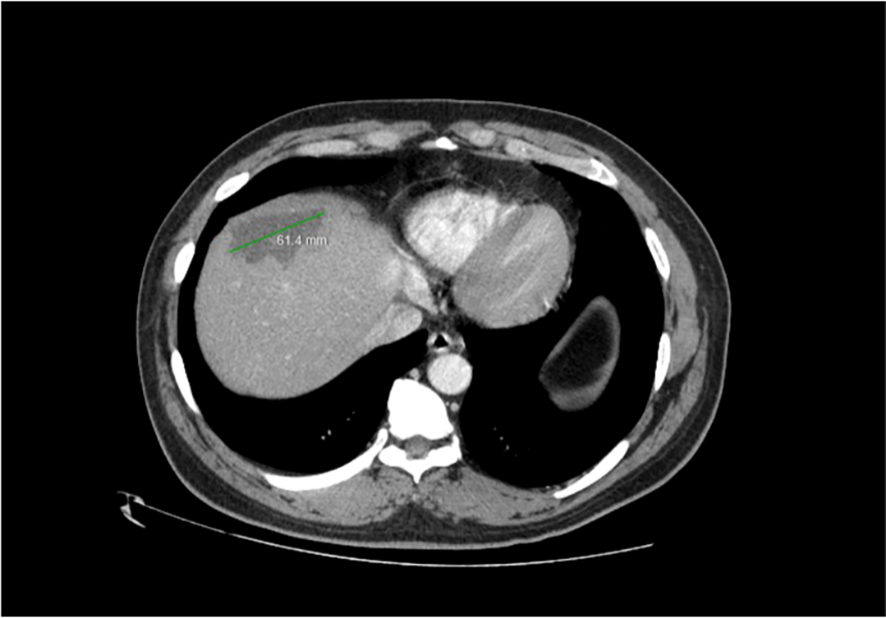
Axial view of CT Abdomen with contrast, showing a 6cm hypodense hepatic lesion, suspected to be metastatic in origin. Satellite lesion not pictured. December 2022.

**Figure 4:**
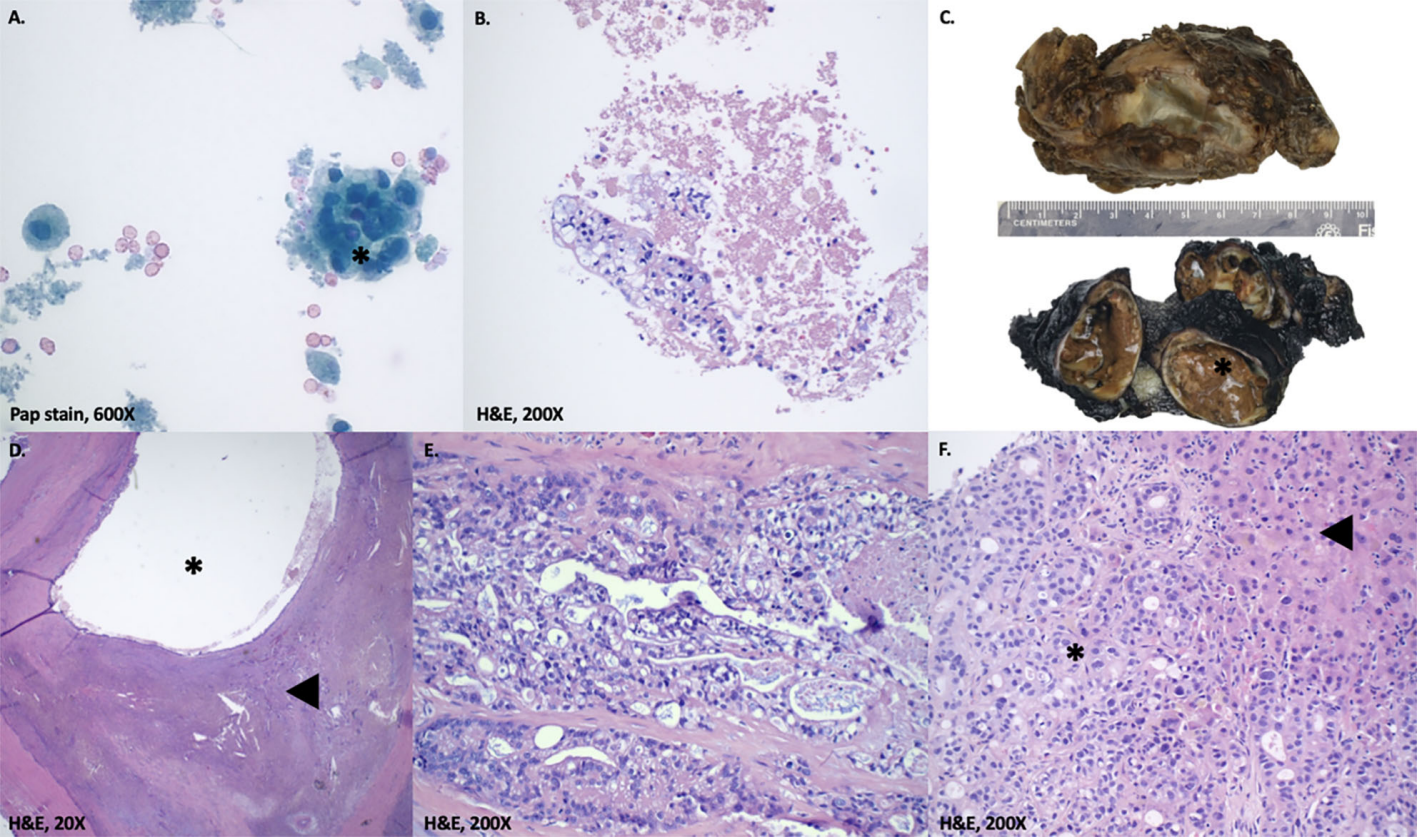
Pathology findings. **(A, B)** Fine needle aspiration of abdominal cyst. Atypical cells with clear cytoplasm arranged in nests(*). Not shown are subsequent immunohistochemical stains on the cell block marking these cells as positive for CK7 and negative for GATA3, PAX8, and OCT3/4. **(C)** Macroscopic gross appearance of retroperitoneal cystic tumor. The specimen measures 8.5 x 6.2 x 4.5 cm, weighs 90 g and upon sectioning, reveals a multilocular cystic mass (*; 6.5 cm largest cyst size) with variably thickened walls. The cysts contain necrotic material and serous-mucoid fluid, with patchy areas of papillary excrescences. **(D, E)** Microscopic appearance of retroperitoneal cystic tumor. D. shows a low-power view of invasive adenocarcinoma (◀) with cystic changes (*). E. shows malignant epithelial cells with cytoplasmic vacuoles and foamy appearance. Not shown are extensive perineural invasion and subsequent immunohistochemical stains marking the tumor cells as positive for CK7, CK19 and negative for CK20, CDX2, GATA3, PAX8, OCT3/4, AFP, Glypican-3, and CD30. **(F)** Liver biopsy. Liver parenchyma (◀) positive for moderately differentiated adenocarcinoma (*). Not shown are subsequent immunohistochemical stains marking the tumor cells as positive for CK19 and negative for CDX2, PAX8, AFP, and CD30.

Imaging of the patient’s retroperitoneal adenocarcinoma and liver metastasis are summarized in **Figures 2**, **3** and **1**, respectively. Additionally, the patient's pathology findings are summarized in **Figure 4**.

### Retroperitoneal adenocarcinoma notable pathology (November 2021)

The pathology of the mass was consistent with a moderately differentiated, invasive adenocarcinoma with cystic changes and extensive perineural invasions. There was no evidence of a germ cell tumor or mesenchymal component of teratoma. Adjacent lymph nodes were negative for carcinoma.

### Liver metastatic adenocarcinoma notable pathology (December 2022)

The pathology of the mass concurred with moderately differentiated adenocarcinoma with necrosis. The overall features suggested a few possibilities and were not specific to a single organ. The histo-morphologic and immunohistochemical profile simulates an adenocarcinoma of pancreatobiliary systems or upper gastrointestinal tract. The possibilities of primary cholangiocarcinoma, metastatic carcinoma from pancreas or upper gastrointestinal tracts should be excluded clinically. The possibility of metastasis from other organs cannot be wholly excluded.

## TEMPUS molecular profiling methods

Tempus xT testing was conducted for both tissue samples. Per Tempus Labs, Inc. Tempus xT (version 4) assay is a custom oncology testing panel consisting of 648 genes with the single nucleotide variants (SNV), insertion and deletion (Indels) events and translocations measured by hybrid capture next-generation sequencing (NGS). A complete list of genes is available at their website. They use DNA extracted from formalin-fixed, paraffin embedded tumor samples. xT-DNA library preparation is performed using IDT unique dual-index adapters, followed by hybrid capture with custom-designed IDT xGen Lockdown probes. The final library is sequenced on an Illumina NovaSeq 6000 instrument. They note their assay has 98.2% sensitivity for SNV above 5% variant allele fraction (VAF), 91.8% sensitivity for indels above 5% VAF, and 91.7% sensitivities for translocations. Tempus xT has been validated (8).

## Discussion

This report describes a case of retroperitoneal adenocarcinoma presenting as lower back and testicular pain years after initial treatment of testicular cancer. One year after his retroperitoneal resection, he presents with an incidentally found liver metastasis. Given the patient’s remote history of testicular tumor, we hypothesize that the subsequent development of retroperitoneal adenocarcinoma represented a metastatic recurrence or somatic transformation of residual retroperitoneal teratoma not fully eradicated by the patient’s prior course of chemotherapy, with the hepatic mass of similar origin. With no access to the prior orchiectomy specimen, this possibility cannot be confirmed. Of note, a very long interval (10-38 years) between the initial diagnosis of testicular teratoma or germ cell tumor and the recurrence as a somatic adenocarcinoma has been reported in the literature (9). Given the lack of other possible origins of the retroperitoneal adenocarcinoma and its position in the “landing zone” of testicular cancer lymphatic metastasis (retroperitoneal lymph nodes), our leading hypothesis is malignant transformation of teratoma, which is a well-described phenomenon in the literature.

Based on TEMPUS analysis, we also hypothesize the newly discovered hepatic mass discovered most recently is a metastasis from the retroperitoneal adenocarcinoma excised one year prior, which is why no primary origin for liver metastasis can be found on PET scan. With this evidence, the patient’s original tumor was probably a teratoma excised decades prior.

Teratoma is a testicular germ cell tumor (TGCT) consisting of cells from two or more primordial germ layers (ectoderm, mesoderm, and endoderm) (10). They are generally benign and have a good prognosis. Mortality can occur due to the somatic transformation into non-TGCT subtypes (e.g., rhabdomyosarcoma, adenocarcinoma). However, this phenomenon is relatively rare, accounting for only 3-6% of all TGCTs (11). The site of malignant transformation of teratoma (MTT) is usually in the primary gonadal tumor or as a metastatic mass in the retroperitoneum. (6)

MTT results in a more aggressive phenotype than non-transformed teratoma. There is only a handful of case series of MTT in the literature, which all report that MTT does not respond to cisplatin-based therapy and that surgery is the mainstay of treatment (12). Rabbani et al. reports that teratoma in the testicular primary tumor is associated with a higher incomplete response rate and residual viable teratoma in the retroperitoneal lymph nodes after primary chemotherapy (13). One explanation for the high chemoresistance of transformed teratomas is that initial platinum-based chemotherapy selectively destroys the chemo-sensitive non-teratomatous germ cells, leaving behind the resistant teratoma and non-germ cell elements. (14)

Interestingly, the literature suggests that MTT develops in 3-6% of chemotherapy-naive patients with metastatic GCT containing teratoma components (6). After platinum-based chemotherapy, the incidence of MTT rises to 14% (15). Using data on 320 patients diagnosed with teratoma with malignant transformation, Giannatempo et al. found that fewer prior chemotherapy regimens were an independent predictor of better overall survival (7). Zeh et al. presented a case of MTT to metastatic retroperitoneal renal cell carcinoma four years after platinum-based chemotherapy for a TGCT with teratomatous components (6). These observations support the chemotherapy-induced selection pressure hypothesis, which argues that tumor cells with favorable mutations for the evasion of chemotherapy are more likely to survive and replicate (16).

We sent our patient’s retroperitoneal adenocarcinoma tissue for TEMPUS molecular profiling to elucidate the genomic etiology of this rare tumor type. Profiling revealed 12 missense mutations that were all variants of unknown significance (VUS) rather than proven pathogenic: *ATP7B, ERCC2, CTNNA1, HIF1A, RAD21, EP300, ABCC3, BCLAF1, NSD1, SEMA3C, FLT1, and TET2 (* **Table 1**). Based on the American College of Medical Genetics (ACMG) guidelines, the classification of genetic variants is a five-tiered scheme that relies on the quantity and quality of evidence needed to classify the variant as pathogenic, likely pathogenic, a variant of uncertain significance (VUS), likely benign, or benign(25). If a variant is classified as a VUS, there is currently insufficient evidence to determine if it is related to a disease.

**Table 1:** List of mutations discovered in TEMPUS testing in retroperitoneal adenocarcinoma with associated VAF and proposed mechanism.

Gene	Function	Variant	Variant allele fraction (VAF)	Possible mechanism
ATP7B	Copper-transporting ATPase 2 protein; export of copper out of cells	c.4232G>A p.R1411Q Missense variant NM_000053	61.5%	Increased platinum efflux out of cancer cells(17)
ERCC2	DNA helicase protein; nucleotide excision repair (NER) pathway	c.491A>G p.H164R Missense variant NM_000400	54.9%	Enhanced DNA repair(17) Cisplatin-induced DNA damage involves the formation of platinum-DNA adducts. The NER pathway is responsible for removal of these bulky adducts. Higher levels of ERCC2 promotes resistance by preventing cisplatin-induced DNA damage and apoptosis of cancer cells. (18)
CTNNA1	Catenin alpha-1; cell adhesion process	c.839A>G p.Y280C Missense variant NM_001903	49.9%	No association with cisplatin resistance found
HIF1A	Hypoxia-inducible factor 1 subunit alpha; master transcriptional regulator of the adaptive response to hypoxia	c.1292C>T p.T431I Missense variant NM_001243084	46.4%	Indirectly *via* upregulation of the expression of platinum resistance related genes: such as signaling genes, DNA repair enzymes, drug efflux transporters, and EMT transcription factors. (17) Increases glycolysis and acidic waste production, which results in increased multidrug transporter expression and cisplatin resistance. Knockdown of HIF-1a reduces cisplatin resistance by redirecting aerobic glycolysis in resistant cancer cells towards mitochondrial oxidative phosphorylation, leading to cell death through overproduction of reactive oxygen species (ROS). (19)
RAD21	Cohesin complex component; Double-strand DNA break repair	c.1349G>A p.R450H Missense variant NM_006265	40.2%	Enhanced DNA repair. (17) As an important protein involved in the process of homologous recombination DNA repair, RAD21 reduces cisplatin-induced double strand DNA damage and cancer cell apoptosis. (20)
EP300	E1A binding protein p300; histone acetyltransferase; regulates transcription *via* chromatin remodeling; Increases activity of p53; HIF1A co-activator.	c.410G>C p.G137A Missense variant NM_001429	34.5%	Prevention of epithelial-to-mesenchymal transition (EMT). EMT is an important step in cancer metastasis involving the loss of E-Cadherin and increased ability to degrade the extracellular matrix and invade other tissues. EMT also generates cancer cells with stem cell-like characteristics. Transcriptional activators of E-Cadherin, such as EP300, help prevent EMT and maintain the cell in an epithelial state. The loss of EP300 is thought to allow for initiation of EMT, generation of cancer stem cells, and thus contribute to drug resistance.(21)
ABCC3	Canalicular multispecific organic anion transporter 2;	c.1975G>A p.V659M Missense variant NM_003786	27.6%	Increased platinum efflux out of cancer cells(17)
BCLAF1	BCL2 associated transcription factor 1; transcriptional repressor; overexpression induces apoptosis	c.472A>G p.R158G Missense variant NM_014739	16.0%	No association with cisplatin resistance found
NSD1*	Nuclear receptor binding SET domain protein 1; enhances androgen receptor transactivation	c.661G>A p.A221T Missense variant NM_022455	15.0% (retroperitoneal adenocarcinoma) 15.0% (liver adenocarcinoma)	Wnt/β-catenin signaling; inhibits apoptosis in cancer cells(17) Overexpression of NSD1 promotes accumulation of β-catenin in the nucleus, which is thought to promote the upregulation of oncogenes, such as c-Myc and cyclin D1. (22)
SEMA3C	Semaphorin 3C;	c.1583G>A p.R528Q Missense variant NM_006379	13.4%	Sema3C enhances invasion and facilitates stem cell marker expression *via* the upregulation of EMT, conferring drug resistance.(17, 23)
FLT1*	FMS related receptor tyrosine kinase 1; member of VEGF family; growth factor signaling	c.3901A>T p.S1301C Missense variant NM_002019	12.9% (retroperitoneal adenocarcinoma) 20.6% (liver adenocarcinoma)	No association with cisplatin resistance found
TET2*	Methylcytosine dioxygenase; chromatin remodeling; DNA demethylation	c.3748G>A p.E1250K Missense variant NM_001127208	12.1% (retroperitoneal adenocarcinoma) 17.3% (liver adenocarcinoma)	Loss of TET2 leads to DNA hypermethylation at enhancer elements, silencing of tumor suppressor genes, and drug resistance.^36^ TET2 causes transcriptional repression of IL-6, an important inflammatory mediator in the tumor microenvironment. Increased levels of IL-6 have been shown to promote multi-drug resistance by activating various pathways implicated in cell cycle regulation and proliferation.(17, 24)

*Found in both retroperitoneal adenocarcinoma and liver adenocarcinoma.

After searching the COSMIC database, only one of the variants (c.4232G>A; ATP7B) has been found in adenocarcinomas - in gastric adenocarcinoma (26). None of the variants have been linked to teratomas in the sparse literature. While none of the other variants are associated with adenocarcinomas in cancer databases, most of the genes have been implicated in various cancers, as summarized in **Table 1**, although not necessarily the exact missense mutation discovered in our case.

Many of these genes have demonstrated a key role in chemoresistance, especially in platinum-based agents such as cisplatin. Huang et al. created a database of over 900 genes associated with platinum resistance over the last 30 years(17). Within the database, 9 of the 12 mutated genes found in our patient were linked to cisplatin-resistance in cancer cells: *ATPB7, HIF1A, ABCC3, SEMA3C, ERCC2, RAD21, CTNNA1, EP300, TET2*. Although the patient’s precise treatment history abroad is unknown, he did likely, in fact, receive cisplatin chemotherapy, as it remains the backbone of chemotherapy regimens for testicular cancer and is prescribed to 10-20% of all cancer patients (17). With a history of a testicular tumor, the presence of these cisplatin-resistance mutations in the patient’s resected retroperitoneal tumor is undoubtedly intriguing. It warrants a broader discussion about the pathogenesis of MTT and its relation to cisplatin resistance, especially in the contemporary era of next-generation sequencing.

## Cisplatin resistance and ATP7B

Platinum-based chemotherapy is a prevalent treatment for various cancers. Platinum-based agents bind and crosslink in GC-rich regions of DNA, which promotes apoptosis *via* the p53 pathway.(27) Several genetic mechanisms of chemoresistance to platinum-based chemotherapy have been postulated, including (1) increased drug efflux or decreased influx *via* ATPase transporters, (2) increased detoxification *via* glutathione, and (3) increased repair of cisplatin-induced DNA adducts (28). The proposed mechanisms of cisplatin-resistance for each of the nine genes are listed in **Table 1**.

One interesting mutation discovered in this patient’s retroperitoneal adenocarcinoma was a 4232G>A Missense mutation in the ATP7B mutation, with a variant allele fraction of 61.5%. This exact missense mutation has also been identified in a case of gastric adenocarcinoma. (26) ATP7B is the gene classically mutated in Wilson’s Disease (WD) (29). Clinically, there is no indication that the patient suffered from WD. However, the variant allele fraction (VAF) for this missense mutation in ATP7B resulted at 61.5%. Generally, a variant is potentially a germline mutation if the VAF is approximately 50% or 100%. (30) While this patient does not suffer from WD, Tempus testing suggests this patient has a germline mutation in ATP7B and could have been identified as high-risk before receiving platinum-based treatment, preventing his eventual MTT and Stage IV cancer diagnosis. It is worth noting that WD results from a defective ATP7B protein, while cisplatin resistance is theorized to be a pathophysiology of overexpression. (31)

ATP7B expression has an established relationship with cisplatin resistance. It has been demonstrated repeatedly that higher expressions of ATP7B are associated with tumor resistance to cisplatin, although the exact mechanism is unknown (32, 33). Dmitriev et al. proposed three mechanisms by which higher levels of ATP7B could result in cisplatin-resistance: 1. active efflux by ATP7B, 2. cisplatin sequestration inside cells *via* the direct binding of platinum to the metal-binding repeats in the N-terminal domain of ATP7B protein causing catalytic phosphorylation of ATP7B, 3. or by an unknown mechanism by which the intracellular copper concentration indirectly influences Cisplatin concentrations and resistance (34)

However, each of these hypotheses has limitations, and the true mechanism for cisplatin resistance remains unknown. For example, at physiological pH, it has been shown that any potential cisplatin efflux by ATP7B would unlikely contribute to drug resistance (34, 35). In support of the theory of a direct binding mechanism, Leonhardt et al. found that mutations in the first 5 N-terminal copper-binding sites of ATP7B did not inhibit the cisplatin-induced catalytic phosphorylation of ATP7B and that deletion of the first four copper-binding sites abolishes the effect of cisplatin on ATP7B activity, suggesting this direct binding of cisplatin to ATP7B plays a role in tumor resistance to cisplatin. (36) Mariniello et al. identified three drugs (Tranilast, Telmisartan, and Amphotericin B) that reduce ATP7B-related cisplatin resistance. (37)

A significant limitation is the unknown impact of the VUS in ATP7B on protein expression or function in our patient. Our discussion is predicated upon the caveat that the true VUS implications are not definitive, making the clinical interpretation of VUS challenging. For one, their effect on protein structure and function is not apparent, making it difficult to classify them as neutral or deleterious. According to ACMG guidelines, a VUS should not be used to guide clinical-decision making. In some cases, a VUS can be reclassified as pathogenic or benign. One study on variant reclassification found that 91% of reclassified variants are downgraded to “benign” and 9% upgraded to “pathogenic” or “likely pathogenic” (38).

However, even VUS or benign mutations may play a role in drug resistance and be deleterious in the proper clinical setting, i.e., following exposure to platinum-based chemotherapy. The current literature supports a possible association between prior cisplatin exposure and the development of MTT, which warrants further investigation in the era of precision medicine.

## The metastatic adenocarcinoma of unknown primary

Regarding the patient’s recent metastatic presentation of liver adenocarcinoma, the primary remains unknown, with the differential including a teratoma undergoing malignant transformation into a retroperitoneal adenocarcinoma further metastasizing to the liver versus a metastasis from a separate unknown primary. Since CT and PET scans revealed no evidence of a primary tumor, the leading hypothesis is that the retroperitoneal adenocarcinoma is the primary site and lead to micrometastatic spread to the liver before being removed.

Notably, the Tempus-xT revealed that the new liver mass involved three of the same mutations in the retroperitoneal adenocarcinoma: *FLT1, TET2, NSD1*. The variants of these three genes are precisely the same for both adenocarcinomas, suggesting that the liver mass is a direct metastasis from the retroperitoneal tumor. Of the three genes, only TET2 – investigated as a tumor suppressor gene in various cancers - has been linked to cisplatin resistance. Zhou et al. demonstrated that cisplatin-resistant cells have much lower levels of TET2 expression compared with non-resistant cells (39). TET2 expression has also been implicated in overall survival in other adenocarcinomas. Nickerson et al. found that decreased TET2 expression in prostate adenocarcinoma is strongly associated with reduced patient survival (40). Deng et al. demonstrated that low TET2 expression predicts poor overall survival in patients with gastric adenocarcinoma (41). Since the TET2 variant is classified as a VUS, it is unclear whether it could result in under- or over-expression of the protein and therefore predict our patient’s survival.

The Tempus RNA-Seq Report, which provides information about mRNA expression levels in a tumor sample, revealed overexpression of CCND1 and under-expression of CDKN2A. Noel et al. found that deregulation of CCND1 and overexpression of cyclin D1 is a major cause of cisplatin resistance in TGCTs(42). Researchers knocked down CCND1 using siRNA and found that combined CCND1 knockdown and cisplatin treatment inhibited cell growth and induced apoptosis *in vitro* significantly more effectively than any single treatment. Overexpression of CCND1 has also been linked to cisplatin resistance in squamous cell carcinomas. (43) The under-expression of CDKN2A, a tumor suppressor gene, has been implicated with poor prognosis in bladder and head and neck cancers.(44, 45) There is no clear evidence that it is mainly responsible for cisplatin resistance. While at the nascent cusp of discovering specific mutation’s role in cisplatin resistance, we hope that classifying our findings can help guide future researchers.

## Conclusions

In the era of precision medicine, treatments are shifting from tumor type- or organ of origin-focused to gene-directed, based on individual biomarker profiling and next-generation sequencing. We do not know if this patient developed cisplatin resistance due to the specific missense mutations discovered in his tumor sample. However, this patient’s story is interesting and raises suspicion about MTT as the leading diagnosis, with cisplatin resistance implicated in many of the mutations discovered in the tissue sample. While we urge caution in making conclusions based on VUS, we find it intriguing and helpful to report their associations with cisplatin resistance. Future research should investigate other genes associated with cisplatin resistance further to understand the pathogenesis of cisplatin resistance for better prediction. In the future, chemotherapy may be more individualized and precisely administered based on germline and tumor mutations.

## Data availability statement

The original contributions presented in the study are included in the article/supplementary material. Further inquiries can be directed to the corresponding author.

## Ethics statement

Ethical review and approval was not required for the study on human participants in accordance with the local legislation and institutional requirements. The patients/participants provided their written informed consent to participate in this study. Written informed consent was obtained from the individual(s) for the publication of any potentially identifiable images or data included in this article.

## Author contributions

MW conceived the idea. CF and EJ conducted most of the research and writing with support from RA and SI. All authors contributed to the article and approved the submitted version.
